# Pittsburgh 1990 Conference–New Products

**DOI:** 10.1155/S1463924690000098

**Published:** 1990


Journal of Automatic Chemistry, Vol. 12, No. 2 (March–April 1990), pp. 80–91

### Pittsburgh 1990 Conference—New Products

*The following were among the many new instruments and services offered through the exposition at the New York meeting*

### High-performance mass spectrometers for organic analysis

In direct response to the explosive growth in the pharmaceutical, biotechnical, and environmental industries, Finnigan MAT has introduced the TSQ 700 triple stage quadrupole and SSQ 700 single stage quadrupole mass spectrometers. These new mass spectrometers, shown at the 1990 Pittcon, are ideal for university, industrial and governmental laboratories—especially where high performance and high productivity are key requirements.

Designed for both MS and MS/MS applications, Finnigan's TSQ 700 is based on the TSQ 70, the industry standard for MS/MS instrumentation, and ensures enhanced performance by incorporating a non-linear octapole collision cell, an advanced 20 kV dynode detector, and a new X-windows-based version of the ICIS data system running on a DECstation 2100 workstation with the ULTRIX-32 operating system (DEC's version of UNIX).

The SSQ 700 is a single-stage version of the TSQ 700, and shares identical inlet techniques and post-acquisition mass spectrometry applications software.

*Advanced data system*

Finnigan MAT now offers an enhanced version of its ICIS data system with the 700 Series mass spectrometers. The ICIS II data system moves Finnigan MAT into the world of UNIX.

A key advantage of the ICIS II data system is that, through the use of the DECstation technology, it provides an easy-to-use multi-tasking, multi-windowing environment that can be mouse or command-line driven to provide the user with maximum speed, without sacrificing the power, features, and flexibility of the original ICIS data system. The 700 Series systems include as standard features: a PostScript laser printer, TCP/IP Ethernet networking protocol, and a complete set of mouse-driven functions, such as window resizing, 'point

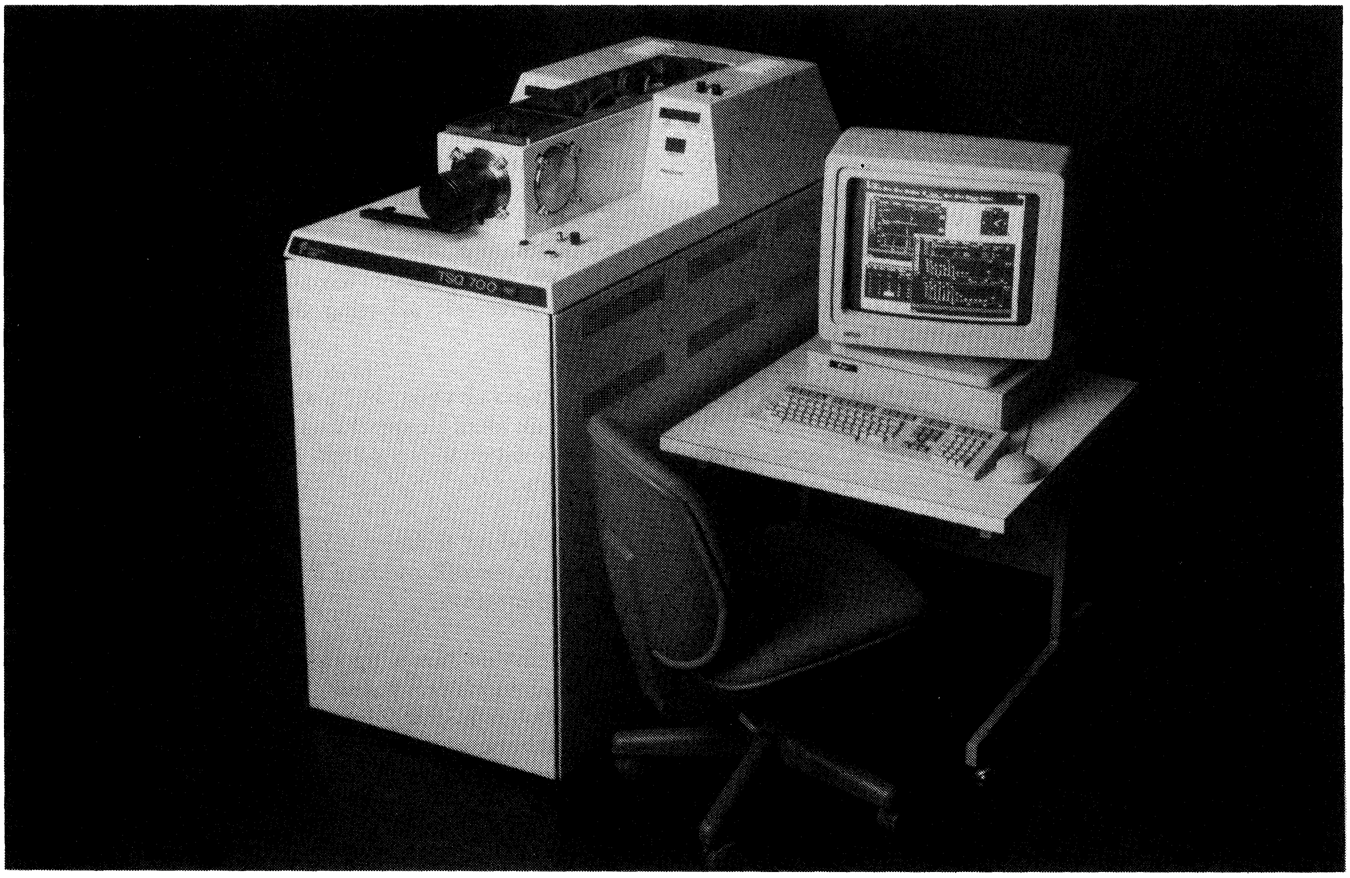

*Finnigan MAT's Triple Stage Quadrupole 700 (TSQ 700), a high-performance GC or LC/MS/MS/DS system is targeted for university, industrial and governmental laboratories. Designed for both MS and MS/MS applications, the TSQ 700 ensures enhanced performance with its X-windows-based version of the ICIS data system running on a DECstation 2100 workstation with the ULTRIX-32 operating system (DEC's version of UNIX).*

and click' selection of scans for spectral display, and library searching and selection of new data files.

*Unique instrument control language*

Finnigan's powerful, easy-to-use, proprietary Instrument Control Language (ICL) allows the mass spectrometer to make real-time data-dependent decisions during acquisition. The ICL can be instructed to optimize the configuration of the mass spectrometer, inlets, lens voltages, and analyser voltages; the acquisition parameters; and the experiments themselves, based on incoming data.

ExperTune, a system-resident set of ICL procedures, automates the tuning and calibration of the mass spectrometer. All MS and MS/MS functions can then be further optimized through the use of simple commands. The user is given the option of modifying system-resident procedures or writing personalized procedures to fully customize and/or automate the mass spectrometer for specific laboratory applications.

*High performance*

The unique non-linear octapole collision cell is a key performance feature of the TSQ 700. It allows increased reproducibility of MS/MS spectra and makes tuning for MS/MS even easier than with a quadrupole or hexapole. The octapole collision cell provides maximum transmission of ions under multiple-collision conditions, while preventing high energy neutrals from reaching the detector. The 20 kV post-acceleration/conversion dynode detector has been designed to be virtually free from electronic noise.

*System versatility*

Both the TSQ 700 and SSQ 700 offer a variety of inlets and ionization techniques. Standard features include: a gas chromatograph (GC), switchable EI/CI ion source with exchangeable ion volumes and beam-collimating magnets, the ability to detect positive and negative ions on alternate scans, a mass range of 10 to 4000 dalton, and a cradle vacuum system with differential turbomolecular pumping.

Inlet techniques include TSP2, a second generation instrument-controlled thermospray interface, fast atom bombardment (FAB), Bio-Probe (a continuous-flow FAB), desorption and solids probes, supercritical fluid chromatography (SFC), and electrospray ionization.

*Details from the Finnigan Corporation, 355 River Oaks Parkway, San Jose, California 95134, USA. Tel.: 408 433 4800; fax: 408 433 4823.*

### Ionization technology for biotechnology and pharmaceutical research

The Electrospray Ionization System (ESI) was also launched by Finnigan MAT at Pittcon 1990. This new ionization technology makes it possible to obtain both molecular weight and structural information for biological and organic compounds at trace levels by combining ESI and innovative data processing software with high performance tandem quadrupole mass spectrometry.

*Target markets*

The Electrospray Ionization System is targeted for the biotechnology, pharmaceutical and academic markets, especially where accurate mass measurement and structure elucidation of biopolymers and other polar molecules is required. The high performance capabilities of the ESI System are also well suited for the general organic and environmental markets.

*Performance*

The new Finnigan MAT ESI System, combined with the TSQ 700 triple stage quadrupole mass spectrometer, is designed for routine determination of molecular weight and structural information for compounds present at picomole or femtomole levels. Taking advantage of ESI's multiple charging capability, molecular weight determination of peptides and proteins can typically be made with 0·02% or better accuracy. In addition, ESI and the TSQ 700 produce structural information at picomole sample levels for peptides.

The ESI System, together with the TSQ 700, also brings to the analyst increased information processing power with the incorporation of the RISC architecture, 32-bit DECstation 2100.

*System versatility*

The ESI System fits directly to the TSQ 700 platform, and is interchangeable with other ionization modes (EI, CI, TSP, and FAB) which allows full flexibility for various types of analysis. This feature allows users of existing TSQ 700 70 mass spectrometers the option of retrofitting ESI.

Electrospray is a valuable adjunct to the sample introduction and ionization capabilities of both the TSQ 70 and 700 Series mass spectrometers. Compounds such as proteins and peptides, drugs and their metabolites, dyes, alkaloids, pesticides and their metabolites, and others that generate ions in solution will benefit from high sensitivity MS and MS/MS analysis with the ESI System.

Samples can be introduced to the ESI System either by liquid chromatography (LC) or by capillary electrophoresis (CE). By simple stream splitting, the effluent from standard 4·6 mm LC columns as well as 2·1 and 1 mm microbore columns can be effectively analysed. For fused silica column LC or CE, the entire effluent is introduced into the mass spectrometer. Utilizing the versatility of the TSQ 700, it is possible to make software directed real-time data dependent decisions during acquisition to decide which parent ion to select for MS/MS analysis. This procedure conserves the sample and generates molecular weight and structural information all in one chromatographic run.

*Software*

The critical link between data generation and problem solving is data interpretation. The following software tools are included with ESI System hardware only on the TSQ 700, as part of the total analytical solution for the biochemistry laboratory:

(1) New algorithms for plotting reconstructed mass chromatograms to further improve signal-to-noise for LC or CE/ESI-MS (for example selected ion reconstructed chromatogram or SIRC).

(2) A deconvolution program (BIO-MASS) which automatically converts the multiply-charged ion envelope plot (% relative abundance versus *m/z*) generated by proteins into a single mass plot (% relative abundance versus mass) from which the accurate molecular weights can be read directly.

(3) A peptide sequence matching program (PEPMATCH) which automatically compares MS/MS daughter ion data for putative known peptides with their predicted MS/MS ions.

(4) Additional software to ease and improve the interpretation of ESI data, as well as data generated by other ionization techniques, particularly as they apply to biochemistry and biotechnology.

*Details from Finnigan MAT (above).*

### High-resolution magnetic mass spectrometry

Also new from Finnigan MAT is the MAT 95. The MAT 95 is a high throughput system with the versatility to perform trace environmental analyses and high mass determinations. The instrument is especially suited to trace-level dioxin analyses.

The MAT 95 brings to the analyst vastly increased information processing power with the incorporation of the RISC architecture DECstation 2100. This 32-bit computer employs the ULTRIX-32 operating system, DEC windows applications, and affords the user maximum flexibility for networking to a variety of peripheral equipment and other data systems.

The MAT 95 offers a variety of inlet systems and ionization techniques. They include: GC, solids probe, fully automated solids probe (AUDEVAP), desorption CI probe, FAB, continuous flow FAB, thermospray LC/MS, FD/FI, and positive and negative chemical ionization.

The MAT 95 can be upgraded to the MAT 95Q tandem mass spectrometer, by the addition of a non-linear octapole collision cell and high-performance mass analyser.

*Details from Finnigan MAT (above).*

### Environmental scanning electron microscope

ElectroScan Corporation announced at Pittcon that its recently introduced environmental scanning electron microscope (ESEM) will soon be used to investigate the degradation process of basic materials making up art and architectural treasures.

The Getty Conservation Institute in Marina del Rey, California, has purchased an ESEM to observe directly how environmental pollutants affect the materials used by painters, sculptors and architects.

Up to now it has been impossible to watch many degradation processes as they occur because the standard scanning electron microscope (SEM) does not display the process while it's happening. According to the Getty, it was previously possible only to get a 'before and after' picture of what takes place to ESEM allows conservationists to view the sample in its natural, unaltered state and to observe and videotape the changes in real time. More can be learnt by combining still micrographs with dynamic information from the video. Conservationists will understand better under what conditions things degrade—stones, metal, pigment—and then be better able to devise controls to preserve them.

*More information from Electro Scan, 66 Concord Stret, Wilmington, Massachusetts 01887, USA.*

### Mass spectrometer

Delsi-Nermag Instruments introduced the RESOLVER3-ES. This triple quadrupole mass spectrometer equipped with the electrospray/ionization source (ESPI) is targeted at the protein/peptide sequencing market. With ESPI, molecules in the Kilodalton mass range can be introduced into a specially engineered collision cell where fragmentation of the multiply charged ions takes place. Data generated and acquired by the PDP-1173 computer is transfered to a small PC where all MW and interpretation calculations are handled. Thermospray and FAB ionization options are also available.

*More information from Delsi-Nermag Instruments, 15701 West Hardy Road, #1, Houston, Texas 77060, USA. Tel.: 713 847 0811.*

### Digital announces ILA standards program enhancements

Digital Equipment Corporation announced Phase III enhancements to its Integrated Laboratory Automation (ILA) Standards program at Pittcon. Phase III of the ILA Standards Program expands the standards to include user interface criteria, as well as enhanced technical criteria for communications. The enhancements make it simpler for laboratory instruments and applications from different vendors to exchange information and integrate their applications.

Although Data Integration levels are not changing, Digital continually enhances components of Data Integration via the Digital CDA (Compound Document Architecture) specifications. All extensions and changes to the ILA Program are evolutionary, requiring no backward changes for present participants. Extensions include enhancements to the quality and levels of data interchange.

In addition to an evolutionary growth in the number of levels, the ILA Standards Program will pursue optional validation testing for user information where practical. While the level requirements for different technical criteria have been expanded, the explanation for conformance has been clarified and simplified.

The ILA Standards Program, a key component of Digital's Computer Integrated Research (CIR) strategy, is the first and only standards program aimed at laboratory integration. It defines how products communicate and share data for applications such as real-time data acquisition, data analysis, laboratory information management, scientific document processing and various other laboratory applications. ILA supports a compatible integrated environment from the laboratory bench to the supercomputer. Products are available from Digital, from third-party laboratory instrument vendors and from software vendors. Currently, more than 36 vendors supply 80 plus conforming products. The goal in 1990 is to expand to 48 vendors and 100 plus products with more than a dozen reaching the new highest level of conformance (class A). Class A conformance provides end-users with seamless application integration requiring little or no effort on the part of customers.

The technical criteria revisions affect two areas: communications and user interface.

*Communication:* with international standards now a reality for communications, the ILA Program was updated to include OSI under DECnet (DECnet/OSI). To provide proper UNIX connections TCP/IP has also been added.

*User interface*: a new component has been added to the technical criteria for user interface. The highest level of support will be DECwindows (XUI or MOTIF). DECwindows, and NAS service, specifies the key components for a consistent end user and application developer interface. These criteria are critical to end users for making their environment easy to learn and use, with a consistent 'look and feel'. Application developers can gain application portability across systems (i.e. ULTRIX, VMS etc.), as well as ensuring a solid application integration environment for the end user.

Digital's Compound Document Architecture is an open, integrated architecture which provides a complete environment for the creation, manipulation, management, exchange, publishing, viewing, mailing, storage, and retrieval of revisable compound documents throughout a networked heterogeneous computing environment. The diverse information types supported by CDA include text, graphics, image, numerical and tabular data. CDA is extensible and will expand to include new technologies, including voice and video.

Digital's Document Interchange Format (DDIF) and Tabular Interchange Format (DTIF) are both part of the Compound Document Architecture. These formats provide a means of storing information so that it can be accessed by any conforming application. With CDA, data can be shared and transported without the need for specific product-to-product data bridges. Compliance with CDA also reduces or eliminates the need for additional programming by end user organizations. Specifications are being pursued for extending the 'Data Interchange' criteria to accommodate analytical instrument data. This extension could be implemented via the Digital CDA tools as an extension to DTIF.

*Details from Richard Gauthier, Digital Equipment Corporation, 4 Results Way MRO4-2/C16, Marlboro, Massachusetts 01752-912, USA. Tel.: 508 467 7752.*

### Gas mixer

Environics Inc., a Connecticut manufacturer of gas management instruments, unveiled its Series 2000 Computerized Multi-Component Gas Mixer, which blends nine gases simultaneously. The instrument permits users to rapidly generate multi-component, precision gas calibration standards by dynamic dilution. Low PPM and PPB concentrations of component gases can be achieved with 1% accuracy. The instrument makes it easy for the analytical chemist to improve the quality of gas analyser data through frequent calibration without expensive, specially prepared cylinders.

The Series 2000 uses the powerful Inmos 'T' series Transputer, a 32-bit parallel microprocessor capable of handling 800 000 floating point instructions per second (FLOPS). This microprocessor commands the S-2000s mass flow controllers in response to user instructions entered through the keyboard. The S-2000 comes equipped with a 25-line, 80-character cold cathode lit LCD display and serial data interfaces.

*Details from Environics Inc., 165 River Road, West Willington, Connecticut 06279, USA. Tel.: 203 429 0077; fax: 203 429 5040.*

### LabStation

LabStation, which was demonstrated in New York, provides the most cost-effective solution for integrating analytical instruments and data with corporate and laboratory computer systems. LabStation is a PC-based, universal solution applicable to all instruments and all LIMS.

For the industrial environment LabStation provides all the features required by quality assurance, production and quality control. LabStation provides a consistent user interface to all instruments. Automated, repeatable methods ensure integrity of tests and data. Communications capabilities provide immediate notification of results. In research and development, pre-clinical and product and process development, LabStation offers other unique strengths. A modular design provides the capability of reconfiguring a workstation. New instruments can be easily added.

In summary, LabStation is a product designed to provide the Laboratory System that you need. From a single workstation, to a building block for a Laboratory System, to the integral component operating under any LIMS or corporate system; LabStation grows with you and continually meets your changing requirements.

*Details from Taratec, 1170 Route 22 East, Bridgewater, New Jersey 08807, USA. Tel.: 201 725 8090.*

### JEOL

The Analytical Instruments division of JEOL USA had several new instruments to introduce in New York City. Their Mass Spectrometry group featured 'COMPLEMENT', a new data system built around the HP's engineering workstation. COMPLEMENT offers a perfect match to the JEOL'S high resolution magnetic sector line of Mass Spectrometers by combining ease of use with very high speed 32 bit computing power.

The NMR group demonstrated for the first time the CPF-400 NMR spectrometer. With the introduction of the CPF-400, the cost of high field NMR is reduced to what was reserved for routine NMR spectrometers. Like the very popular CPF-270, the CPF-400 is capable of being operated by the non-expert in a full automation mode, but still has the ability to produce high quality research data.

Also at Pittcon JEOL USA announced a new line of instruments; the RE series of ESR spectrometers. These very high sensitivity instruments have been a tremendous success in the rest of the world. In addition to being used in analytical laboratories, the RE series has found great popularity in the newly emerging biochemical applications.

*Full details from JEOL USA, 11 Dearborn Road, Peabody, Massachusetts 01960, USA. Tel.: 508 535 5900.*

### Chemist's access system

Molecular Design Ltd launched the Chemist's Access System, an add-on enhancement application to its existing set of programs, the Chemist's Personal Software Series (CPSS). The Chemist's Access System will let researchers automatically log-on to and search the online chemical databases CAS Online, Beilstein File, and Beilstein Online, via the search services STN International or Dialog, and to subsequently use retrieved data in their CPSS programs on a PC.

ChemTalk Plus is CPSS's program for terminal emulation, file transfer and online database access, designed specifically for chemists. Using ChemTalk Plus enhanced by the Chemist's Access System, users will be able to logon automatically with the click of a mouse to CAS Online or Beilstein File via the search service STN International, or to Beilstein Online via the search service Dialog. Once logged onto CAS Online chemists can perform chemical structure or substructure searches of the CAS Registry File or text searches of the CA File with a simple mouse command. Or, logged onto Beilstein File or Beilstein Online, chemists can perform chemical structure or substructure searches of these databses, again with a simple mouse command. In either case, because they draw the structure queries offline in ChemTalk Plus prior to logging on, expensive online time is minimal. The Chemist's Access System automatically translates structure queries into a form that STN International's CAS Online and Beilstein File or Dialog's Beilstein Online understands, so chemists need only know the drawing conventions of their PC software.

Offline, users can easily transfer the captured information into a ChemText document or into a ChemBase database. ChemText is CPSS's image and text processor for chemists and ChemBase is CPSS's chemical database management system for individual chemists. Users do not need to redraw structures or retype data.

When transferring captured data to a ChemText document, the Chemist's Access System transfers both the structure graphics and the textual information into ChemText. When transferring data to a ChemBase database, it automatically places fields of the originating database, such as Registry Number, Common Name, Molecular Formula, and Molecular Model Information into corresponding ChemBase data fields for future searching. The system places structural images from Dialog's Beilstein Online into molfile fields that can be structurally searched. It places structures from STN International's CAS Online and Beilstein File into metafile data fields. Although not searchable by structure (as are structures entered directly into ChemBase), these images round out the database with a graphic view of the molecular data.

*Further information from Molecular Design, 2132 Farallon Drive, San Leandro, California 94577, USA. Tel.: 415 895 1313.*

### Sulphur chemiluminescence detector

The Model 350B Sulfur Chemiluminescence Detector for GC and SFC was introduced by Sievers Research. The Model 350B is based on the same proven chemistry as the Model 350 but incorporates new electronics to provide a wider linear range of greater than five orders of magnitude, reduced background noise and no measurable peak broadening. The Model 350B can be fitted to any GC or SFC equipped with a flame ionization detector. Both the sulphur signal from the SCD 350B and the hydrocarbon signal from the FID can be monitored simultaneously without post-column splitting. The Model 350B is the most sensitive sulphur-selective detector available for use with both GC and SFC.

*Details from Sievers Research, Inc., 1930 Central Avenue, Suite C, Boulder, Colorado 80301, USA. Tel.: 303 444 2009; fax: 303 444 9543.*

### Particle size analyser

A new line of MICROTRAC(R) particle size analysers that can measure particle sizes in the ultrafine range (down to 0·005 microns), as well as sizes up to 700 microns, with one modular system, was demonstrated by Leeds & Northrup.

Designated the Series 9200, the new line provides the broadest size range in the most compact system of any other known offering on the market. The basic MICROTRAC system (see picture) comprises: (1) a laser-based analyser, selected from a choice of three models for the range desired; and (2) a Computer Control Module, which has extensive software to drive the particle size analyser in either an 'Operating' mode or a 'Data Management System' mode.

The Series 9200 system can measure a variety of materials—fine or coarse, wet or dry, in small or large sample volumes. For research, testing, or production control applications, the laser-based MICROTRAC has already been proven effective for literally thousands of materials—such as cement, ceramics, chemicals, explosives, foods, oil/water emulsions, pharmaceuticals, talc, and tar sands.

For a minimum initial investment, the user can start with one of three analysers, each of which works with the same Computer Control Module. They are: *Full range analyser,* (0·1 to 700 microns); *Standard range analyser,* (0·7 to 700 microns); and *Ultrafine particle range analyser* (0·005 to 3 microns). Each system can cover its full range with a single measurement using the latest in light-scattering laser technology. Tests can be run as fast as one a minute to provide reliable, repeatable results.

Compactness of the Series 9200 System is evident from the photograph which shows two of the three available analysers on either side of the Computer Control Module. The Ultrafine particle analyser (UPA) on the left measures just 4 × 6 × 15 inches, and the Full range analyser (FRA) on the right measures 22 × 12 × 13 inches. The Standard range analyser (SRA) is identical in size to the FRA. All three with the Control Module can easily fit on a 3 × 5 foot bench.

*Details from Leeds & Northrup, North Wales, Pennsylvania 19454, USA. Tel.: 215 699 2000.*

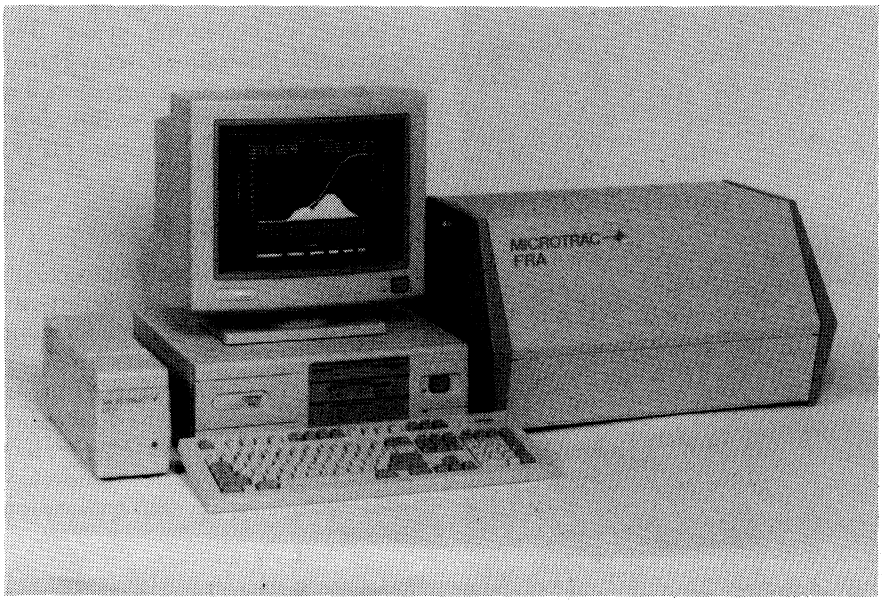

*The Leeds & Northrup MICROTRAC Series 9200 Particle Size Analyser comprises a (1) Computer Control Module (centre), which uses a fast and reliable, MS/DOS-based Compac 386; and (2) a selected model of analyser. On the left is the UPA model with a 0·005 to 3 micron range, and on the right, the FRA model with a 0·1 to 700 micron range. A third analyser option, the SRA for a 0·7 to 700 micron range, is similar in appearance and size to the FRA.*

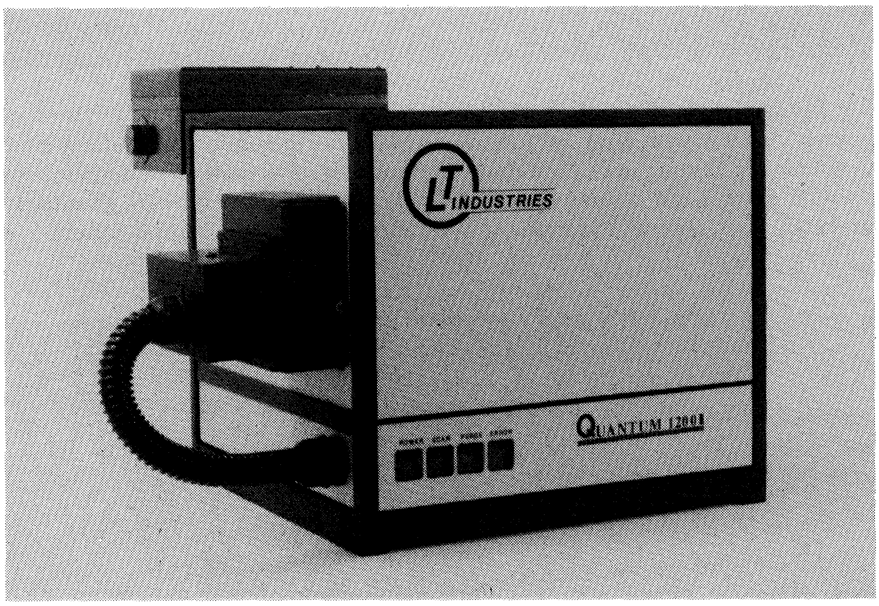

*The Quantum 1200I PLUS, the industrial version of the Quantum 1200 PLUS VIS/NIR General Purpose Analyzer, which was designed for long-term unattended use in process environments including hazardous environments. The instrument, which was demonstrated at the 1990 Pittcon, performs real-time non-destructive analysis of 10 or more constituents, simultaneously. It operates on the process line on a real-time basis (five full spectrum scans per second). Details from L.T. Industries, 6110 Executive Blvd, Rockville, Maryland 20852, USA. Tel.: 301 468 6777.*

### Quiet vacuum pumps

Galileo Vacuum Systems has introduced a new set of quiet, portable rotary vacuum pumps for a variety of industrial and scientific applications.

The small pumps provide a good ultimate vacuum, low working temperature and are light in weight for heavy duty use with displacement from 4 CFM to 57 CFM. The 'Vacsound' line of pumps is equipped with a built-in lubricating pump for optimum operating safety and high pumping speed at all working pressures from 1000 mbr down. Pumps come with either single or three-phase American motors and feature hydraulically operated isolation valves to avoid pressure rise or suction line contamination.

High gas ballast flow gives the pumps increased capacity to handle condensable gases and water vapour. They have no copper or copper alloy parts and employ fluorinated elastomer gaskets. They are constructed for economical service, having interchangeable parts, and the entire inner pump module can be easily replaced.

*For further information contact Galileo Vacuum Systems, a division of Galileo Corporation of America, at 64 Field Road, Unit-3B, PO Box 868, Somers, Connecticut 06071, USA. Tel.: 203 763 4004.*

### Introductions from Waters at the Pittsburgh Conference

Waters Chromatography Division of Millipore introduced a capillary electrophoresis system, a pulsed electrochemical detector for high sensitivity chromatographic applications, a photodiode array detector for improved spectral analysis and column chemistries for HPLC at the 1990 Pittsburgh Conference in New York City. These new products are described briefly below.

*Waters Quanta 4000 Capillary Electrophoresis System*

Waters new, affordable Quanta 4000 (see picture) is a fully automated capillary electrophoresis system that allows multiple, unattended analyses and offers an autopurge feature that speeds throughput and improves accuracy by automatically cleaning the capillaries after each run. The Quanta 4000 is compatible with Waters chromatography workstations and features a high-sensitivity, low noise UV/Vis detector designed to detect even trace level components. Waters Quanta 4000 Capillary Electrophoresis System can be used to separate biomolecules, pharmaceuticals, and small ions.

*Waters 991 Photodiode Array Detector*

The Waters 991 Photodiode Array Detector offers improved spectral analysis capabilities providing immediate qualitative assessment of peak homogeneity and rapid distinction of spectal differences and similarities. The 991 also provides automated peak purity checking for the detection of co-eluting peaks. The Waters 991 Photodiode Array Detector is available as a free-standing module or as part of a fully-integrated Waters PowerLine HPLC system.

*464 Pulsed Electrochemical Detector*

The Waters 464 Pulsed Electrochemical Detector offers high sensitivity for a broad range of chromatographic applications. It offers three modes of operation, DC, Pulse and Scan, to provide versatility for the analysis of a variety of compounds. The Waters 464 offers a variety of cell and electrode options for specific application chemistries as well.

*Enhanced Software for Waters Maxima and Baseline Chromatography Workstations*

The Version 3.3 of the Maxima and Baseline Chromatography software allows calibration of non-consecutive peak groupings for analysis of PCBs

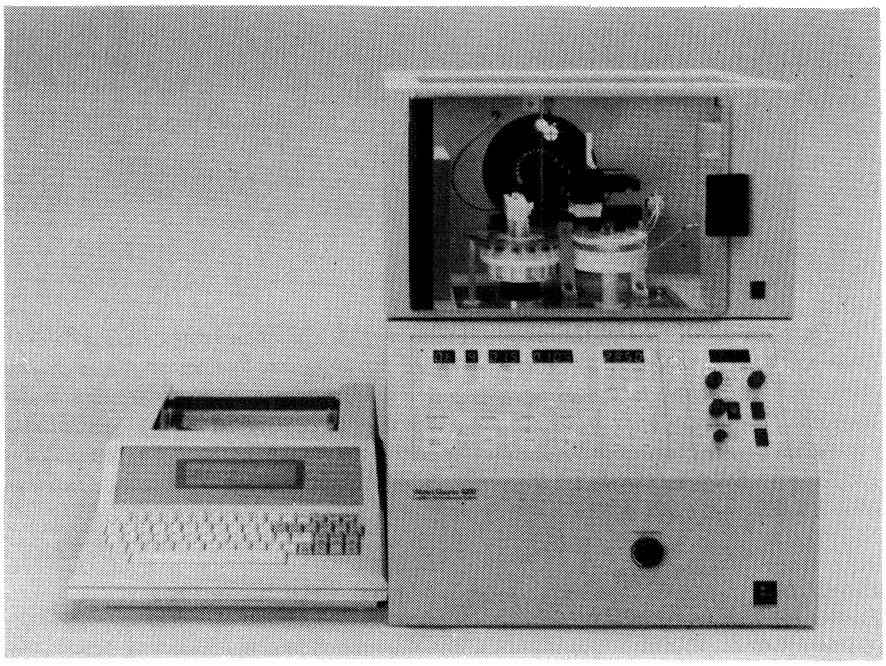

*Waters Quanta 4000 Capillary Electrophoresis system.*

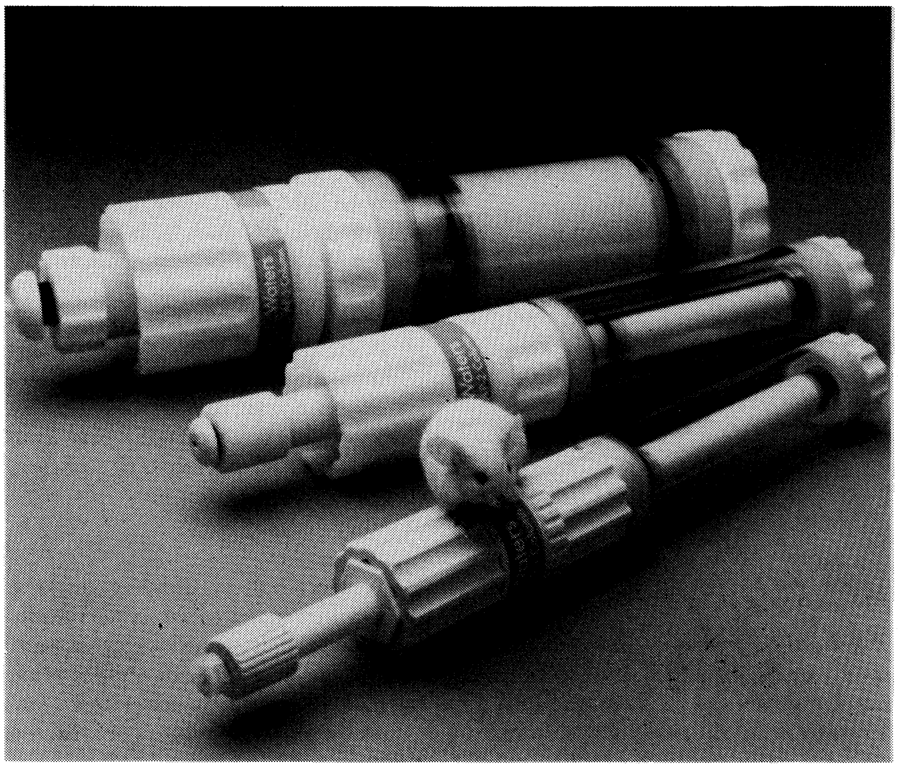

*Waters Protein-Pak HR (high performance resin) ion exchange columns.*

and other multi-peak analytes. Other improvements include integration with multiple sets of integration parameters for better results on multiple detector systems, 24-pin print resolution for improved graphics on dot-matrix printers, and enhanced LC and QC system control capabilities.

*Enhanced GPC Option for Maxima Chromatography Workstations*

This Gel Permeation Chromatography (GPC) software performs broad standards (Purdon-Mate) calibration, in addition to the narrow standards technique. New graphic program capabilities include the clear definition of baseline and processing start and end points and new screen displays include area and molecular weight normalized distributions and overlays of chromatograms.

*New Cartridges and Chemistries*

In addition, Waterss showed a new line of high resolution, prepacked columns for the analytical and preparative separation of biomolecules. Waters Protein-Pak HR (see picture) (high performance resin) ion exchange columns permit direct purification of immunoglobulins from ascites or cell culture supernatants. The Protein-Pak HR series provide high resolution, excellent recovery of protein mass and biological activity and high protein binding capacity. Performance is optimized when used with Waters 650 Advanced Protein Purification System.

Waters also announced a non-metallic Delta-Pak $C_{18}$ column. This 3·9 × 150 mm column is packed with 300 Å, 4 μm reverse phase material. The Delta-Pak column is compatible with Waters non-metallic 625 LC System and allows the selection of novel mobile phase modifiers such as HCl for reversed phase HPLC peptide mapping. Use of HCl modified mobile phases is made possible by incorporating polymeric materials in the advanced Delta-Pak and 625 LC System designs.

*For more information on any of these products please contact Carole Wade-Clark at Waters Chromatography Division of Millipore, 34 Maple Street, Milford, Massachusetts 01757, USA.*

### Automated sample preparation

Among the many products demonstrated by Zymark in New York was the range of Workstations which are designed to eliminate such routine laboratory sample preparation tasks as dilutions, reagent and internal standard additions, vortex mixing, weighing, membrane filtration, solid phase extractions, and dual HPLC auto injection. The BenchMate precisely and accurately performs dilutions either gravimetrically or volumetrically and can even determine the density of your sample. Methods are set up through use of simple menu screens using a non-dedicated PC. An audit trail is created using sample ID number and weight values. This data is stored on disk and can be presented in a simple spread sheet format. The BenchMate Workstation uses industry standard disposable filters and solid phase extraction columns.

*Details from Sharon Correia, Zymark Corporation, Hopkinton, Massachusetts 01748, USA.*

### Computer-controlled distillation device

Bran + Luebbe Analyzing Technologies showed a new TRAACS 800 microdistillation module at PittCon which automatically analyses cyanide, phenol and other volatile analytes in drinking-water, sea-water or industrial waste water twice as quickly than previous automatic methods and 25 times faster than conventional manual means.

The module uses EPA Certified reference check samples to analyse up to 50 samples per hour with an accuracy fully equivalent to manual methods with much lower detection limits and significantly higher levels of consistency and repeatability

Bran + Luebbe's advanced computer-controlled TRAACS 800 wet chemistry laboratory analysis system is a multi-application system in which a computer assumes control of many activities, including sophisticated calibration adjustment, random access sampling and resampling, and even dilution and re-runs of off-scale samples, without any need for operator/manual intervention.

TRAACS 800 is a continuous-flow analytical system with a state of the art peristaltic pump, using electronically controlled pressurized bubble injection for reliable stream segmentation. It has conservative reagent flow rates of only 400–900 μl per minute, up to 10 pump lines per analytical channel, and a new fibre optic dual-channel colorimeter with improved detection limits.

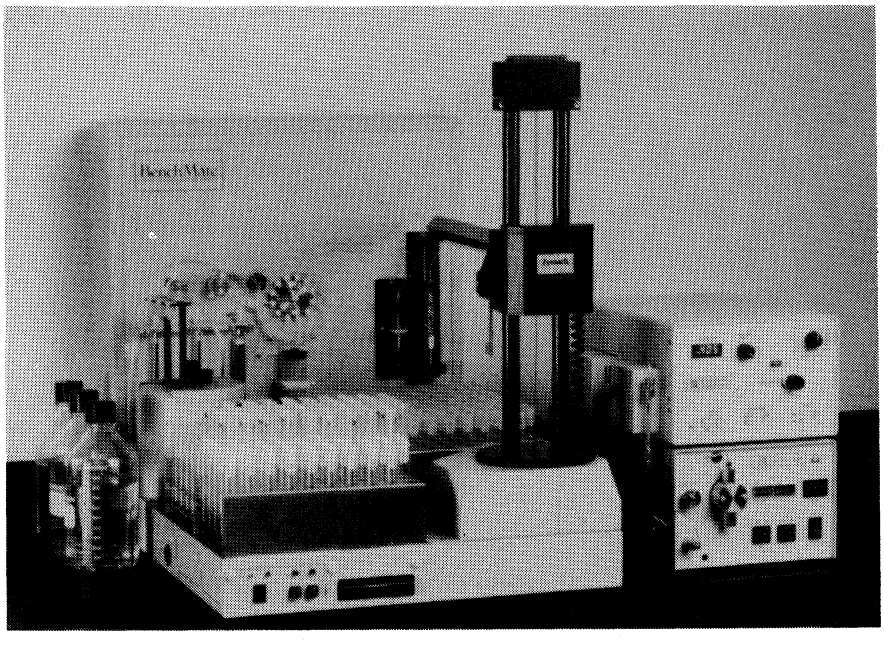

*A Zymark Benchmate workstation—demonstrated at Pittcon 1990.*

The Reagent Sequencer, an advanced option of the TRAACS 800 system, was also demonstrated live at Bran + Luebbe's Pittcon booth this year. In conjunction with TRAACS 800 Environmental Multitest Cartridges, the Sequencer makes it easy for a laboratory to run to 10 separate chemistries in one day without manual changeover. Clean-up and new reagent introduction are accomplished completely automatically, directed by computer.

Bran + Luebbe personnel and key users presented papers at Pittcon on the microdistillation concept, a case history of reservoir eutrification studies in New Jersey and developments in autodilution via the TRAACS 800 system, as well as techniques for stack gas analysis using the Bran + Luebbe On-Line Monitor.

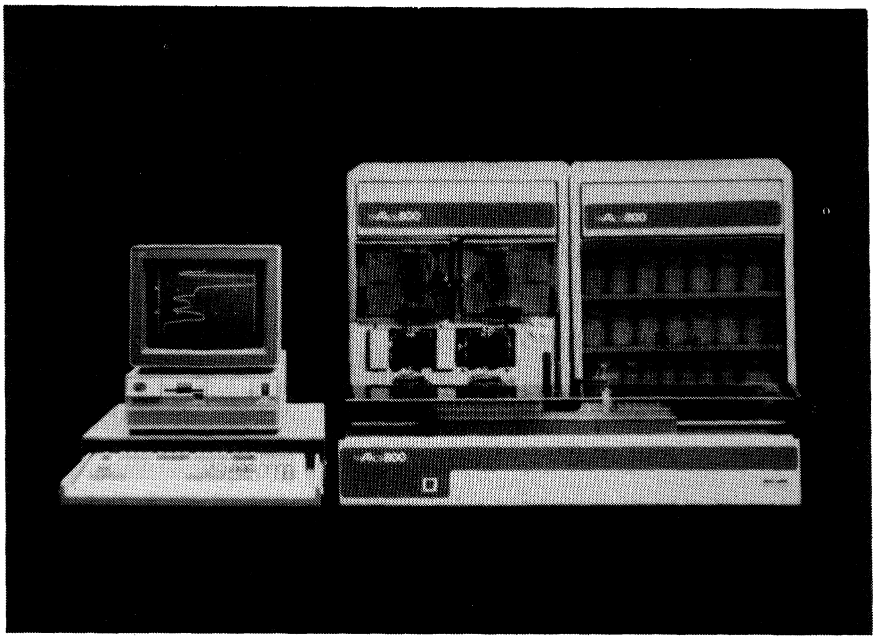

*The Microdistillation Module operates under the control of the Bran + Luebbe TRAACS 800 System shown here.*

In addition to environmental testing, TRAACS 800 is valuable for use in the fields of chemical process, food technology, agricultural testing, toxicology, pharmaceutical quality control, biotechnology and veterinary science.

*Details from Bran + Luebbe, Analyzing Technologies, Inc., 103 Fairview Park Drive, Elmsford, New York 10523-1500, USA. Tel.: 914 524 8133; fax: 914 524 8294.*

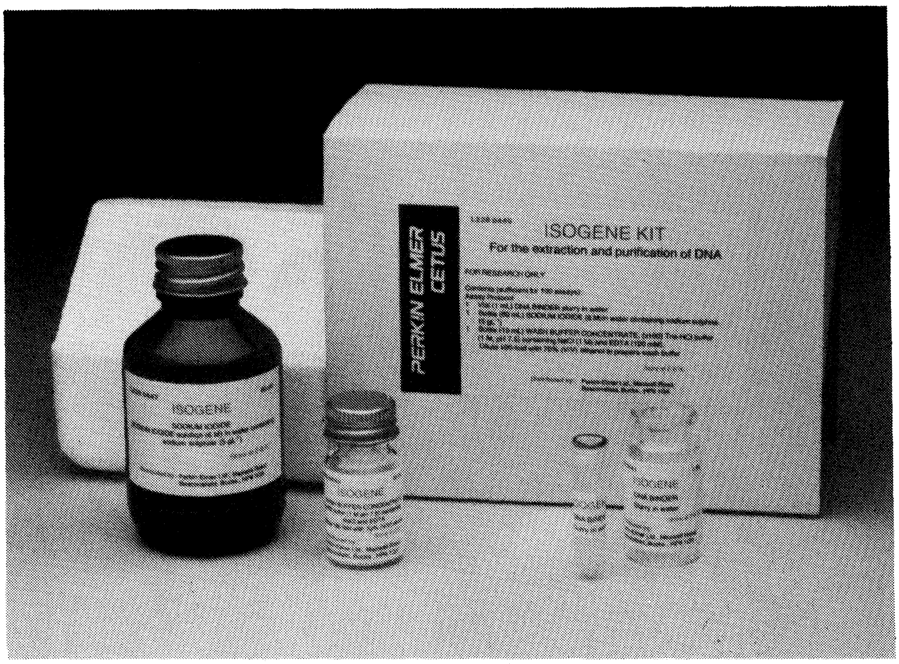

*The new Isogene Kit from Perkin-Elmer Cetus allows bioresearchers to extract and purify sample DNA from impurities that normally inhibit the GeneAmp PCR process. It can also be used to purify PCR-amplified DNA isolated by agarose gels. (Details from Perkin-Elmer.)*

### Perkin-Elmer

Perkin-Elmer announced a substantial number of new products at Pittcon 1990—a few are described here and the rest will be included in later issues of *Journal of Automatic Chemistry*.

### *MGA 1600*

Perkin-Elmer's programmable MGA 1600 Multiple Gas Analyser provides users complete keyboard control through an IBM or Epson Computer for all process gas analysis and other industrial gas monitoring applications. The new MGA can analyse up to 16 components in up to 50 streams, with a typical analysis time of 12 s per stream.

This enhanced version of the MGA 1600 is menu driven and offers five modes of operation. The 'Normal' mode automatically sequences sampling from stream-to-stream. 'Manual Mode' allows the user to intervene the automatic cycle for port specific analysis needs. An 'Investigative Scan' mode allows the entire system to be scanned for any component with mass-to-charge ratios from 2 to 120 making reprogrammability easy. Other system parameters can be adjusted such as: sampling sequences and alarm levels

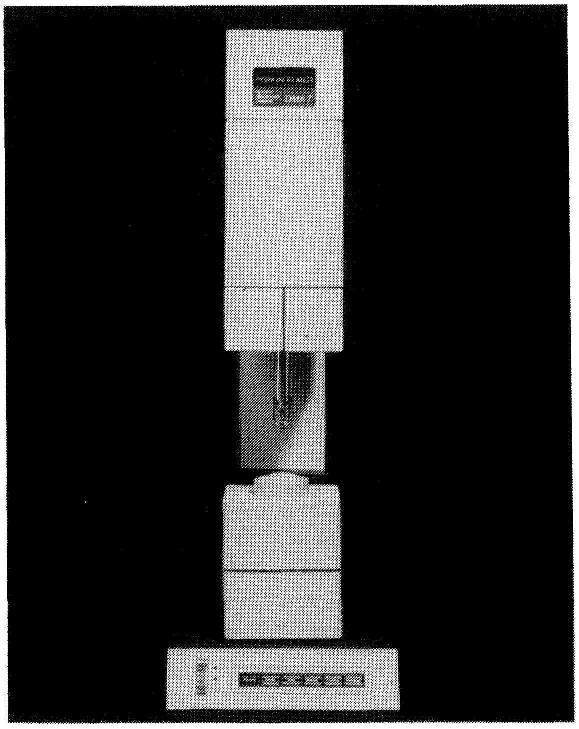

*A new mechanical testing analysis system available from Perkin-Elmer simplifies dynamic testing of a broad range of materials. Called the Perkin-Elmer DMA 7 Dynamic Mechanical Analyzer, the system combines new software and hardware features that allow users to take full advantage of its capabilities for materials characterization, from soft samples to high modulus composites and ceramics. (Details from Perkin-Elmer.)*

via an 'Auxillary mode'. Self-diagnostics prevent inaccurate analysis from being reported due to system malfunction, and a 'Diagnostics mode' will assist in determining the source of error. Automatic calibration and verification (initiated on a timed basis or on command through the keyboard, calibration takes only a few minutes). To verify calibration, a 'check gas' with all the components in the analyses can be automatically introduced.

A magnetic sector mass spectrometer, at the heart of every MGA 1600, ensures accuracy, reliability, and long-term stability. The MGA 1600 can be linked via a modem to Perkin-Elmer's service hot line, thus ensuring application assistance for the most complex system problem without any delay.

*Library search application software for ICAMS*

ICAMS is a Perkin-Elmer toxic gas security system that has become the accepted system of choice for industrial air-quality monitoring applications world wide.

The New Library Search Application Software expands ICAMS chemical identification capability by allowing users to quickly identify 'unknown' chemical compounds in mixtures or unusual odours not found on the ICAMS pre-programmed monitoring list. A list of probable chemicals can be generated to help identify these 'unknowns'. The Library Search Application Software does this in less than a minute. By promptly identifying common invading odours, savings in lost work time and unnecessary plant evacuations can be realized. This capability also helps protect employees from truly hazardous substances by identifying their real nature.

Library Search is an application software program that resides in an ICAMS Remote Analysis Station (RAS). It consists of mass spectral data on more than 30 000 chemical compounds. This large Reference Library is derived from the National Bureau of Standards (NBS) Library. The program also accommodates up to 51 user-defined libraries and has the capability to search this library at a speed of 5000 compounds per minute. Users have a choice of three modes: 'Library Edit', 'Library Search' and 'Library Build' when using the software.

The Remote Analysis Station (RAS) is an IBM PS/2 Series Computer Station and operating Software that allows data archiving and remote operation of up to 99 ICAMS.

Via a user-friendly scheduling utility in the RAS software, operators are able to easily establish an automatic routing for ICAMS data output downloading the compression.

The Remote Analysis Station offers industrial hygenists and environmental engineers a collection and application program computer workstation and provides the link for interaction with various ICAMS data stations throughout a facility. Plant safety and industrial hygiene personnel can view displayed data as well as send and receive information and commands from one central station.

*For more information on Perkin-Elmer products contact; The Perkin-Elmer Corporation, Applied Science Division, N. Garey Avenue, Pomona, California 91767, USA. Tel.: 714 593 3581; fax: 714 596 2301.*

### Lab management and data automation products

Automated Compliance Systems, Inc. (ACS) is a full service environmental data management services company founded in 1988. Headquartered in Bridgewater, New Jersey, ACS has satellite locations in Oak Ridge, Tennessee, Irvine, California, and San Mateo, California. Planning, building, loading, maintaining and managing decision support environmental data bases are ACS's core services. At Pittcon '90 ACS introduced a solution for LIMS (laboratory information management systems) specifically designed for industrial and commercial environmental laboratories.

ACS differs from commercial LIMS solutions in the following ways:

(1) ACS conducts an extensive analysis of the current information processes with each client and produces a detailed performance specification and implementation plan which serves as the 'blueprint' for the system implementation. The result of this effort, the LAB-PLAN (details enclosed), provide the basis for the company's guarantee and defines the requirements unique to each laboratory. This allows ACS to bend its system, not the laboratory and guarantee performance results.

(2) ACS has developed two primary software modules, SEEDPAK1 and SEEDPAK2. SEEDPAK1 is a production, management and control application uniquely adapted to each environmental laboratory. SEEDPAK2 automates the collection, consolidation, and reporting functions within the environmental lab. Together they form a fully integrated application for the management of an environmental laboratory.

(3) ACS's products are based on the standard features of the industry's leading relational database management system (RDMS), ORACLE, and the industry standard fourth generation reporting language, Structured Query Reporting Language (SQL). The applications run in the UNIX environment and are scaleable and portable from 386-based IBM compatible PC's to larger mainframes. The ACS applications are hardware vendor independent. The company provides the source code as part of client licenses.

*Further information from Automated Compliance Systems, Inc., 245 Highway 22 West, Bridgewater, New Jersey 08807 USA. Tel.: 201 707 4100.*

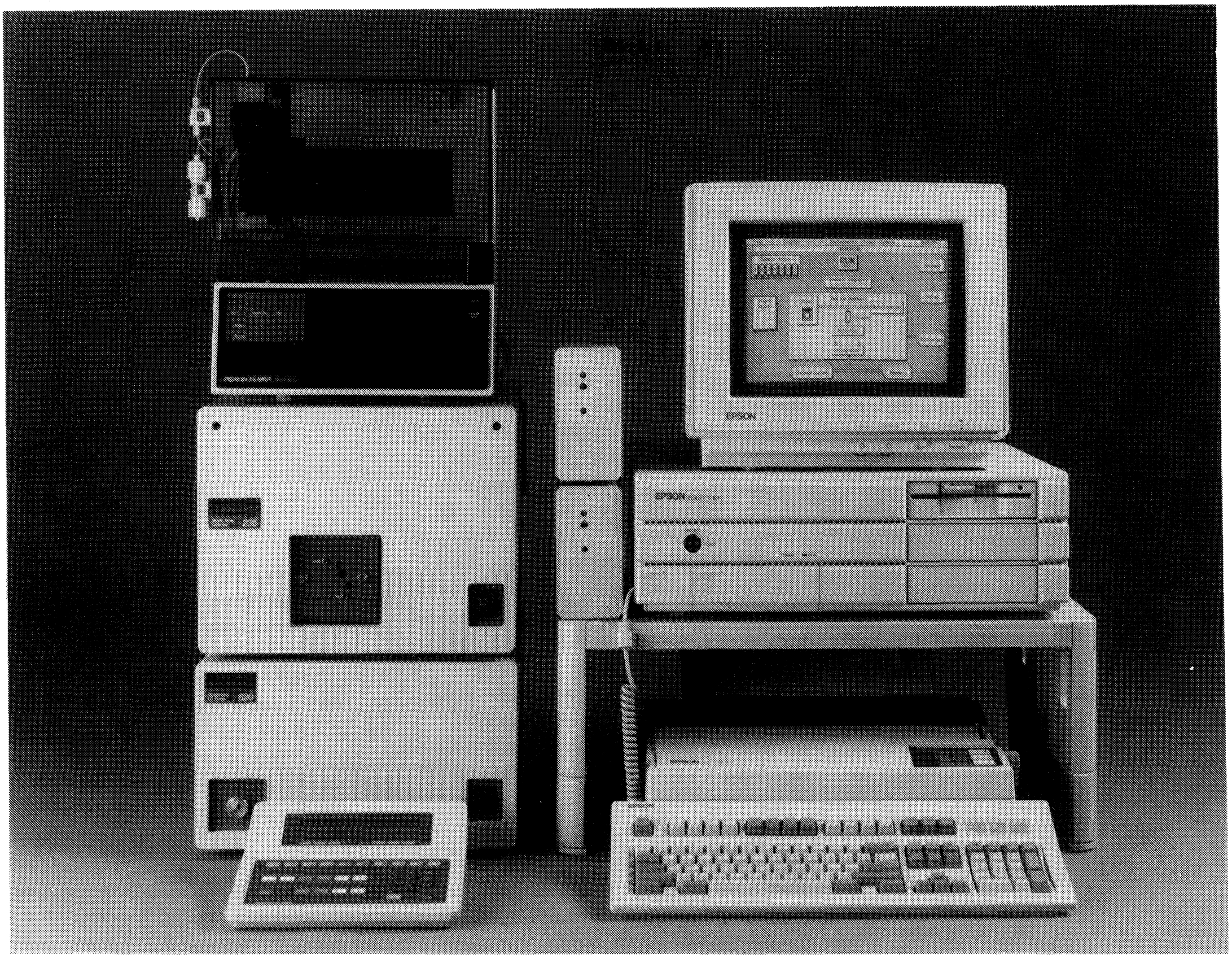

*Representing a powerful combination of HPLC instruments and all of the software necessary for developing separations, the new LC Analyst Expert Methods Development System from Perkin-Elmer has been developed with the requirements of pharamceutical methods developments in mind, as well as to serve as a top-of-the-line research liquid chromatography system for environmental, food and beverage, and other analytical applications. System components include the Perkin-Elmer 620 Quaternary Pump, ISS-200 Autosampler, and LC-235 Diode Array Detector. (Details from Perkin-Elmer.)*

### British instruments

Fifteen British manufacturers demonstrated their products through a booth organized by Gambica, the UK's trade association for the instrumentation, control and automation industry. Some of the companies are described briefly here.

*Kariba Instruments* (Star Technology Centre, Hadfield Road, Leccwith Industrial Estate, Cardiff, Wales CF1 8AQ, UK) exhibited for the first time in the US both an advanced air oven (AAO) for HPLC and an advanced gas chromatograph (AGC). Both utilize a novel heating/cooling approach to achieve sub-ambient and elevated temperatures previously not readily available to HPLC and GC users.

The AGC range of gas chromatographs has been specifically designed so that they can be customized to become 'black box' components analysers for use in dedicated laboratory and process monitoring applications. Both the AAO and AGC reflect the user requirement for minimum footprint (approximately 4″ and 11″ bench width respectively), maximum safety and ease of use.

*Carbolite Furnaces* (Bamford Mill, Bamford, Sheffield 530 2AU, UK) introduced a range of tube furnaces. Examples of rapid heating, high temperature and small chamber furnaces were also demonstrated. The tube furnaces, covering a temperature range from 900°C to 1600°C in a variety of standard tube diameters and lengths, have been designed for use in gas analysis, material testing, ceramic firing, continuous strip heating and thermo-couple calibration. Most of these are available in vertical or horizontal format.

The latest Universal HPLC Detector, the model CD1000 featured by *Analinkl* (Unit D, Cardiff Workshops, Lewis Road, East Moors, Cardiff CF1 5EG, UK) is claimed to be the most effective for detecting any unknown non-UV absorbing compound with gradient elution HPLC. The CD1000 can handle aqueous, non-aqueous and gradient elution at variable flow rates and is able to quickly detect compounds regardless of their functional group. It is claimed to detect unknown and unexpected compounds which have a mass range between 200 and 10 million without having to calculate individual detector responses and it has a simple analog/integrator output.

Several innovative products were demonstrated by *Hi-Tech Scientific* (Brunel Road, Salisbury, Wiltshire SB2 7PU, UK). The Hi-Mix range of sample handling/mixing devices were featured. These allow rapid, precise and easy mixing of reagents and samples for a range of applications including FT-1R, UV/VIS spectroscopy, fluorescence, and rapid mixing techniques such as stopped-flow. Direct injection of mixed components into the cell incorporates accurate thermostatting, and facilitates the handling of hazardous material, even under anaerobic conditions.

*Omnifit* (51 Norfolk Street, Cambridge CB1 2LE, UK) presented new high performance chromatography columns, accessories and fittings, together with miniature inert valves and connectors and solvent delivery systems.

